# Regnase-1 in microglia negatively regulates high mobility group box 1-mediated inflammation and neuronal injury

**DOI:** 10.1038/srep24073

**Published:** 2016-04-05

**Authors:** Xiao-Xi Liu, Chen Wang, Shao-Fei Huang, Qiong Chen, Ya-Fang Hu, Liang Zhou, Yong Gu

**Affiliations:** 1 Department of Neurology, Nanfang Hospital, Southern Medical University. Guangzhou, Guangdong 510515, P. R. China; 2 Department of Neurology, the Second Affiliated Hospital & Yuying Children’s Hospital, Wenzhou Medical University. Wenzhou, Zhejiang 325000, P. R. China

## Abstract

Extracellular high mobility group box 1 (HMGB1) has been demonstrated to function as a proinflammatory cytokine and induces neuronal injury in response to various pathological stimuli in central nervous system (CNS). However, the regulatory factor involved in HMGB1-mediated inflammatory signaling is largely unclear. Regulatory RNase 1 (Regnase-1) is a potent anti-inflammation enzyme that can degrade a set of mRNAs encoding proinflammatory cytokines. The present study aims to determine the role of Regnase-1 in the regulation of HMGB1-mediated inflammatory injury in CNS. Cultured microglia and rat brain were treated with recombinant HMGB1 to examine the induction of Regnase-1 expression. Moreover, the role of Regnase-1 in modulating the expression of inflammatory cytokines and neuronal injury was then investigated in microglia by specific siRNA knockdown upon HMGB1 treatment. Results showed that HMGB1 could significantly induce the *de novo* synthesis of Regnase-1 in cultured microglia. Consistently, Regnase-1 was elevated and found to be co-localized with microglia marker in the brain of rat treated with HMGB1. Silencing Regnase-1 in microglia enhanced HMGB1-induced expression of proinflammatory cytokines and exacerbated neuronal toxicity. Collectively, these results suggest that Regnase-1 can be induced by HMGB1 in microglia and negatively regulates HMGB1-mediated neuroinflammation and neuronal toxicity.

A well-controlled immune response is beneficial to maintaining central nervous system (CNS) homeostasis. When dysregulated and exaggerated, neuroinflammation can lead to significant tissue damage of CNS^1^. A growing number of studies indicate that neuroinflammation has been highly involved in pathologic processes of many CNS disorders including stroke^2^, traumatic brain injury^3^ and neurodegenerative disease^4^,^5^,^6^. Thus, the regulatory factors that modulate neuroinflammation may be beneficial for therapeutic strategy, as well as for a better understanding on the immunopathology of inflammation related CNS diseases.

Danger-associated molecular patterns (DAMPs), known as alarmins, which signal tissue and cell damage are critical for the induction of innate and adaptive immune response, resulting in the production of sterile inflammation^7^,^8^. High mobility group box 1 (HMGB1) has been known as one of the crucial members of DAMPs. It normally locates in the nucleus. Once pathogens or tissue injury occurred, HMGB1 can be either passively released from injured tissue cells or actively secreted by immune cells to extracellular milieu. Subsequently, HMGB1 binds to pattern recognition receptors on immune cells and triggers the intracellular signal cascades, resulting in a robust inflammatory response^9^. In CNS, the release of HMGB1 has been found in a variety of disorders such as stroke^10^,^11^, traumatic brain injury^12^, Alzheimer’s disease^13^,^14^, Parkinson’s disease^15^,^16^ and multiple sclerosis^17^. The extracellular HMGB1 binds to receptors for advanced glycation endproduct, toll-like receptor (TLR)-2, TLR-4 or Mac1, on microglia or infiltrated macrophages. The binding of HMGB1 to its receptors then recruits myeloid differentiation factor 88 to activate mitogen activated protein kinase (MAPK); subsequently, it induces nuclear factor-κB (NF-κB) to start the transcription of inflammatory cytokines, which leads to brain cell damage^15^,^18^,^19^. The activated microglia and injured neurons, in turn, cause further HMGB1 release to trigger an autocrine signaling and contribute to severe inflammatory neuronal and vascular injury. Thus, a vicious cycle is reinforced to aggravate disease outcome.

Intensive studies on the proinflammatory role of HMGB1 have been emerged, however, negative regulation signaling involved in HMGB1-mediated inflammatory pathway remains unclear. Regulatory RNase 1 (Regnase-1), also known as Zc3h12a and monocyte chemotactic protein-1 (MCP-1) induced protein-1 (MCPIP1), is a novel CCCH-type zinc finger motif-containing protein which has endonuclease activity. The purified Regnase-1 can specifically decay a set of cytokine-encoding mRNAs such as interleukin (IL)-6, interferon-γ, IL-1β, IL-12β and IL-2 by recognizing the stem-loop structure in the 3′-untranslational terminal region of these mRNAs^20^,^21^,^22^,^23^,^24^. Stimulation by MCP-1, lipopolysaccharides (LPS) and IL-1β^25^,^26^,^27^ can induce a rapid and potent transcription of Regnase-1 through NF-κB or MAPK^21^,^28^. In CNS, Regnase-1 has been reported to participate in electroacupuncture-induced ischemic stroke tolerance and minocycline-mediated neuroprotection against ischemic brain injury^29^,^30^. Regnase-1 also involves in LPS preconditioning-induced ischemic stroke tolerance by regulating the expression of proinflammatory cytokines^31^. More importantly, suppression of Regnase-1 by microRNA(miR)-9 enhances inflammatory response in microglia^32^. These findings collectively suggest that Regnase-1 can be induced by inflammatory milieu and functions as a regulatory factor to ameliorate neuroinflammatory injury in CNS. Given that MAPK and NF-κB pathways are shared processes of HMGB1-induced inflammation and the production of Regnase-1, we hypothesize that Regnase-1 can be induced by HMGB1 to elicit a negative feedback mechanism which limits the HMGB1-mediated inflammation and neuronal injury.

In this study, we designed series of experiments to testify this hypothesis and found that purified recombinant HMGB1 could induce the expression of Regnase-1 in microglia *in vitro* and *in vivo*. Furthermore, knockdown of Regnase-1 in microglia enhanced transcription of IL-1β, IL-6 and exacerbated HMGB1-mediated inflammatory injury to neurons.

## Results

### HMGB1 induces the expression of inflammatory cytokines in BV2 cells in a dose-dependent manner

**Figure 1:**
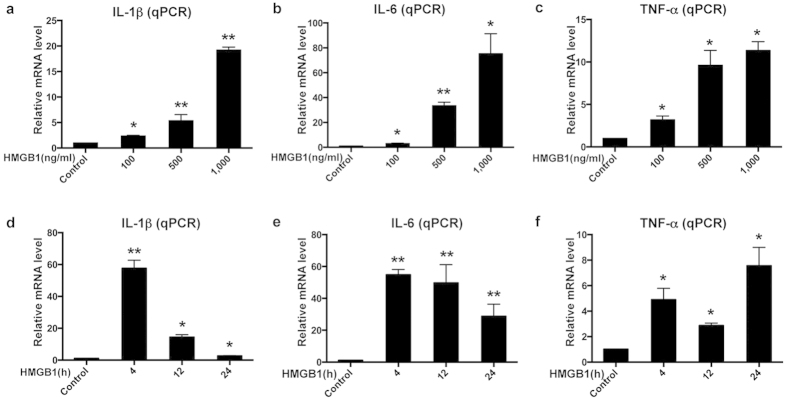
HMGB1 induced the expression of proinflammatory cytokines in BV2 cells. (**a–c**) Relative mRNA levels of IL-1β (**a**), IL-6 (**b**) and TNF-α (**c**) in BV2 cells treated with different doses (100, 500 and 1,000 ng/ml) HMGB1 for 24 h were determined by quantitative real-time PCR (qPCR). (**d–f**) Relative mRNA levels of IL-1β (**d**), IL-6 (**e**) and TNF-α (**f**) in BV2 cells treated with 1,000 ng/ml HMGB1 for different incubation times (0, 4, 12 or 24 h). The relative changes were represented as mean ± SD. n = 3. *p < 0.05, **p < 0.01 versus Control.

To determine the response of BV2, a mouse microglia cell line, to HMGB1 stimulation, we firstly detected inflammatory cytokine levels upon the treatment of recombinant HMGB1 protein. Expectably, HMGB1 did increase mRNA expression of IL-1β, IL-6 and TNF-α in a dose-dependent manner (100 to 1,000 ng/ml) at 24 h. (Fig. 1a–c). Time course studies showed that 1,000 ng/ml HMGB1 significantly up-regulated mRNA expression levels of IL-1β and IL-6, and peaked at 4 h (Fig. 1d–e), while the increase of TNF-α reached the peak at 24 h and presented a biphasic pattern (Fig. 1f). These results indicated that BV2 cells were highly reactive to HMGB1.

### Regnase-1 expression is increased by HMGB1 treatment *in vitro*

**Figure 2:**
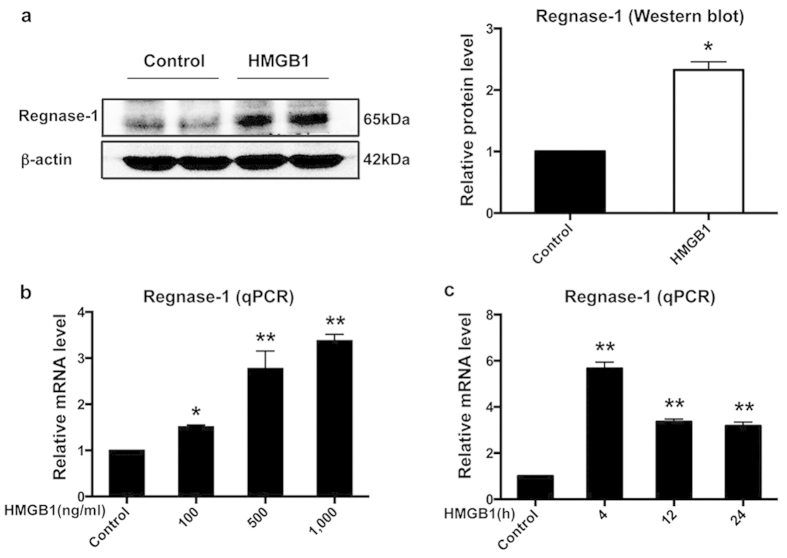
Regnase-1 expression was increased by HMGB1 treatment in BV2 cells. (**a**) Regnase-1 protein expression in BV2 cells with or without HMGB1 (1,000 g/ml) treatment was measured by Western blot. The relative protein level was normalized with β-actin and the quantitative data were represented as bar graph. n = 3. *p < 0.05 versus Control. Full-length blots are presented in supplementary information (see Supplementary Fig. S1). The gels have been run under the same experimental conditions. (1) (**b–c**) Different doses and incubation times of HMGB1 treatment on BV2 cells were employed to observe the mRNA dynamic expression of Reganse-1. The relative levels of Regnase-1 mRNA expression in BV2 cells treated with 100, 500 and 1000 ng/ml HMGB1 for 24 h (**b**), or with 1000 ng/ml HMGB1 for 0, 4, 12 and 24 h (**c**) were determined by qPCR. The relative changes were represented as mean ± SD. n = 3. *p < 0.05, **p < 0.01 versus Control.

We then wondered whether Regnase-1 was involved in the inflammatory response caused by HMGB1. Protein and mRNA expressions of Regnase-1 in BV2 cells were examined by Western blot and qPCR, respectively. As shown in Fig. 2a, there was a low but detectable Regnase-1 protein level in BV2 cells. However, a significant increase of Regnase-1 expression (2.3-fold) was observed after 1,000 ng/ml HMGB1 treatment for 24 h (Fig. 2a). This was further confirmed by employing different doses and incubation times of HMGB1 treatment. Results showed that mRNA level of Regnase-1 increased in a dose-dependent manner following HMGB1 treatment in BV2 cells for 24 h (Fig. 2b). Meanwhile, compared with the control group, 1,000 ng/ml HMGB1 treatment for 4, 12 and 24 h also increased Regnase-1 mRNA expression in BV2 cells (Fig. 2c).

### Regnase-1 expression is induced in microglia by HMGB1 *in vivo*

**Figure 3:**
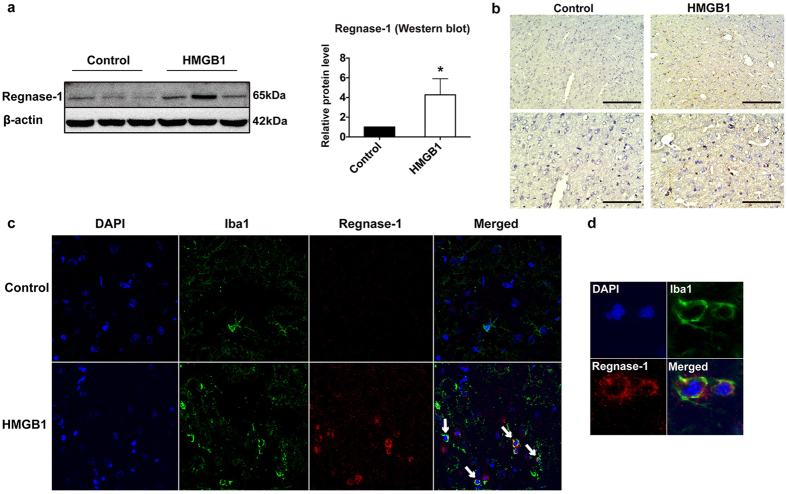
Regnase-1 expression was increased by HMGB1 treatment in rat brain. Rats were randomly divided into two groups and treated with either saline (Control) or HMGB1 (8 μg/kg) for 24 h. (**a**) Regnase-1 protein levels in the brain of rats were determined by Western blot. The quantitative data of relative protein levels were represented as mean ± SD and shown in bar graph. n = 3. *p < 0.05 versus Control. Full-length blots are presented in supplementary information (see Supplementary Fig. S2). The gels have been run under the same experimental conditions. (**b**) The representative immunostaining images showing Regnase-1 immuno-response in rat brain (upper panel: scale bar = 200 μm; lower panel: scale bar = 100 μm). (**c–d**) Immunofluorescent images were captured with confocal micrographs and the white arrows in representative images indicated the co-localization of Regnase-1, Iba1 and DAPI (**c**) Magnification 60×; (**d**) Magnification 480×).

We next investigated whether Regnase-1 was also inducible *in vivo* in response to HMGB1 treatment in rats. HMGB1 or vehicle saline was injected to the right side lateral ventricle. Brain proteins and coronal sections were obtained 24 h after the treatment to observe the protein expression and localization of Regnase-1. Results revealed that Regnase-1 protein level in rats from HMGB1-treated group was much higher (approximately 4.3-fold) than vehicle administration (Fig. 3a). Consistently, immunohistochemistry and immunofluorescence assays showed more intensive staining of Regnase-1 in HMGB1 treated group rats than vehicle control (Fig. 3b–c).

In addition, we determined the anatomical location of elevated Regnase-1 by co-staining microglia in rat brain. Immunofluorescence analysis showed the co-localization of Regnase-1 and Iba1 (the microglia marker), indicating a specific response of microglia to HMGB1 *in vivo* (Fig. 3c–d). These data demonstrated that Regnase-1 expression was induced in microglia by HMGB1 in rats.

### Knockdown of Regnase-1 in microglia increases proinflammatory cytokines expressions

**Figure 4:**
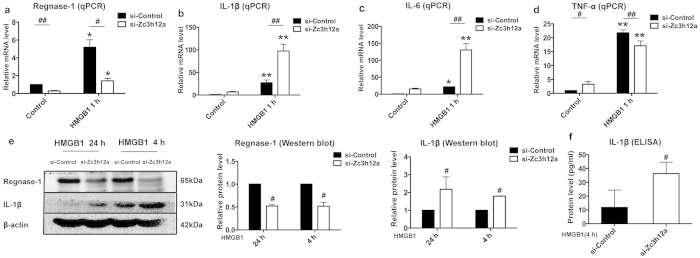
Knockdown of Regnase-1 in BV2 cells increased proinflammatory cytokines expression. BV2 cells were transfected with either siRNA Negative Control (si-Control) or siRNA specific to Regnase-1 (si-Zc3h12a) and then treated with HMGB1 (1,000 ng/ml) or vehicle control for 1 h. The silencing efficiency of si-Zc3h12a to Regnase-1 (**a**) and relative mRNA levels of IL-1β (**b**), IL-6 (**c**) and TNF-α (**d**) were examined by qPCR. (**e**) BV2 cells transfected with si-Control or si-Zc3h12a were treated with HMGB1 (1,000 ng/ml) for 4 h or 24 h. The protein levels of Regnase-1 and IL-1β were evaluated by Western blot. Full-length blots are presented in supplementary information (see Supplementary Fig. S3). The gels have been run under the same experimental conditions. (**f**) After HMGB1 treatment (1,000 ng/ml) for 4 h, the protein level of IL-1β in the culture medium collected from BV2 cells transfected with si-Control or si-Zc3h12a were determined by ELISA. Quantitative data were represented as mean ± SD and shown in bar graph. n = 3. *p < 0.05, **p < 0.01 versus Control; ^#^*p* < 0.05, ^##^*p* < 0.01 versus si-Control.

To assess the role of Regnase-1 in HMGB1-induced inflammation, we transfected BV2 cells with siRNA specific to Regnase-1 (si-Zc3h12a) or control siRNA (si-Control) followed by detection of inflammatory cytokines. We tested three siRNA fragments targeting Zc3h12a and found one could effectively reduce the mRNA level of Regnase-1 under HMGB1 treatment background (Fig. 4a,e). In BV2 cells without HMGB1 stimulus, knockdown of Regnase-1 did not induce obvious mRNA expression change of proinflammatory cytokines (Fig. 4b–d). Upon HMGB1 treatment, the expressions of all three cytokines were much enhanced; IL-1β and IL-6, two putative substrates of Regnase-1, were further increased in Regnase-1-silenced BV2 cells. Interestingly, TNF-α was not increased, but decreased slightly with statistical significance upon Regnase-1 silencing suggesting that TNF-α may not be the direct target of Regnase-1 (Fig. 4b–d). Western blot and ELISA analysis showed that Regnase-1 knockdown promoted IL-1β protein expression and secretion after HMGB1 treatment in BV2 cells (Fig. 4e–f). Collectively, our data suggested that Regnase-1 negatively regulated the expression of proinflammatory cytokines in microglia.

### Conditioned medium (CM) collected from cultured microglia with Regnase-1 silencing is more toxic to neurons under HMGB1 treatment

**Figure 5:**
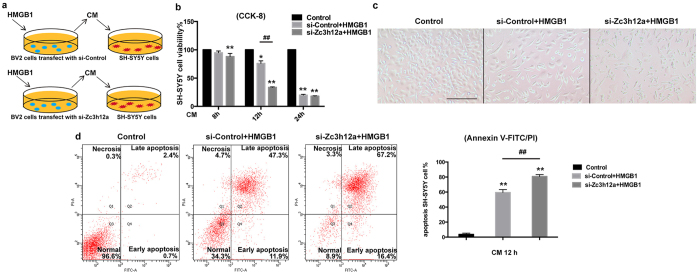
CM collected from BV2 cells with Regnase-1 silencing was more toxic to neurons than control under HMGB1 treatment. (**a**) CM were collected from HMGB1-treated BV2 cells transfected with either siRNA Negative Control (si-Control) or siRNA to Regnase-1 (si-Zc3h12a), and then incubated with SH-SY5Y cells for 8 h, 12 h or 24 h. (**b**) The viability of SH-SY5Y cells was determined by CCK8 assay. (**c**) Representative images of SH-SY5Y cell morphology after 12 h CM incubation were observed under light microscope. Scale bar = 200 μm. (**d**) After incubation with CM for 12 h, SH-SY5Y cells were collected and stained with Annexin V-FITC / PI followed by detection with flow cytometry. The rate of apoptotic cells was calculated. The percentages were represented as mean ± SD. n = 3. *p < 0.05, **p < 0.01 versus Control; ^##^*p* < 0.01 versus si-Control.

To confirm the effect of Regnase-1 on regulating neuronal survival during HMGB1-mediated inflammation, we finally added BV2 CM to SH-SY5Y cells, a neuroblastoma cell line with neuron-like properties, followed determination of neuronal cell toxicity and activity. CM from HMGB1-treated BV2 cells with or without Regnase-1 knockdown was collected to incubate SH-SY5Y cells (Fig. 5a). The viability and apoptosis of SH-SY5Y cells were determined by CCK-8 and Annexin V-FITC/PI assay, respectively. As shown in Fig. 5b, incubation of Regnase-1 knockdown microglia CM with SH-SY5Y cells for 12 h, but not 8 and 24 h led to lower cell viability than control silencing. This was further supported by the morphological changes (Fig. 5c). Annexin V-FITC/PI staining assay was conducted at 12 h after CM treatment and showed that knockdown of Regnase-1 greatly enhanced the cytotoxicity of CM to SH-SY5Y cells (Fig. 5d). These data suggested that the reduction of Regnase-1 in microglia is toxic to neurons.

## Discussion

Extracellular HMGB1 is a well-known cytokine that plays an important role in multiple pathologies. To our best knowledge, studies on regulatory factors involved in the downstream of HMGB1 are rare. In this study, we demonstrated that HMGB1 could induce the expression of Regnase-1 in microglia both *in vitro* and *in vivo*. The loss-of-function of Regnase-1 augmented HMGB1-mediated inflammation and exacerbated inflammatory neuronal injury.

The established proinflammatory role of HMGB1 was confirmed in our research. In agreement with previous studies, HMGB1 treatment induces sharp elevation of cytokines expression. Time course studies indicated mRNA expression of TNF-α displayed a biphasic pattern after HMGB1 treatment. Likewise, a published study indicates TNF-α mRNA production also presents a biphasic pattern after HMGB1 stimulation in human peripheral blood monocytes^33^. Furthermore, HMGB1 activates more TNF-producing monocytes during the later peak^33^. Thus, it is possible that the proliferation of TNF-producing cells is one of the reasons that explains the second peak of TNF-α expression by HMGB1. From the perspective of molecular mechanism, several recently identified factors, such as miR-124, miR-26, miR-146a, miR-155 and TIPE2, have been reported to negatively regulate the TNF-α production during microglia activation^34^,^35^,^36^,^37^,^38^. Besides, TNF-α has been proved to induce a biphasic activation of NF-κB, the transcription factor triggering TNF-α gene expression^39^. Whether these mechanisms contributing to the biphasic expression pattern of TNF-α under HMGB1 treatment in our study are still unknown and deserve further investigation.

HMGB1-induced mRNA increase of IL-1β and IL-6 was peaked at 4 h, however, mRNA encoding cytokines is dynamically regulated at post-transcriptional stages and the protein expression may display a different expression pattern. A previous study reveals that 24 h HMGB1 treatment-induces the highest level of TNF-α and IL-1β in the culture medium of neuron-glia cultures^15^. Thus, 24 h-treatment of HMGB1 was selected to test dose-response of inflammatory cytokines and Regnase-1 in order to remain consistent with the duration of HMGB1 treatment *in vivo* in our subsequent studies.

**Figure 6:**
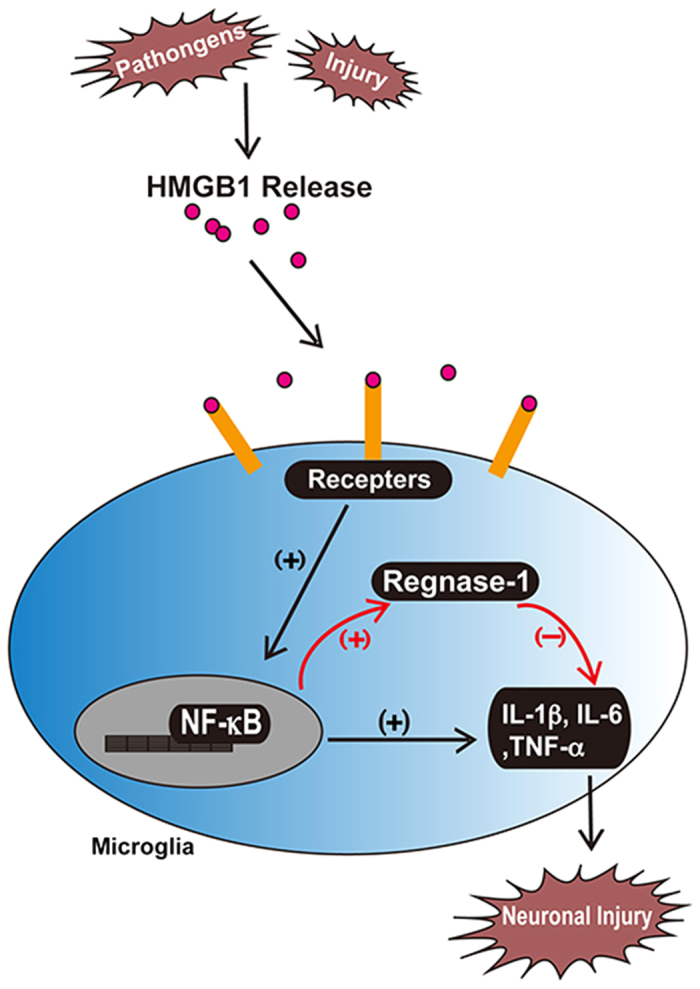
The summarized diagram shows Regnase-1 acts as a downstream regulator during the process of HMGB1-mediated inflammation. In response to pathogens or CNS tissue injury, HMGB1 is actively or passively released to the extracellular milieu and binds to receptors on microglia surface. The activation of receptors then induces the translocation of NF-κB and transcription of proinflammatory cytokines, such as IL-1β, Il-6 and TNF-α. Meanwhile, anti-inflammatory protein Regnase-1 can also be induced by HMGB1 and degrades cytokine-encoding mRNA, restricts HMGB1-mediated inflammation and neuronal injury, serving as a negative feedback mechanism to limit the exaggerated neuroinflammation.

Approaches targeting different processes of HMGB1-induced inflammation are explosively increased in past several years. For example, lysine deacetylation^40^,^41^ and heat shock protein 72 42 inhibit translocation and secretion of HMGB1; HMGB1-binding heptamer suppresses HMGB1 co-stimulation with binding partners^43^; neutralizing antibody abolishes the binding and activation of HMGB1 receptors^44^; some small molecule compounds, for example glycyrrhizin, can non-specifically inhibit multiple steps of HMGB1-induced inflammation^45^,^46^. However, publications regarding to intracellular downstream signaling that restricts HMGB1-mediated inflammatory injury are seldom. Among limited examples, CD13 is reported to negatively regulate HMGB1-TLR4 signaling to maintain the equilibrium of HMGB1-mediated inflammation^47^. Here, we step forward to identify another novel endogenous mediator, Regnase-1, which restrains HMGB1-mediated neuronal injury by fortifying the negative regulatory feedback loop and fine-tuning inflammation pathway to limit the exaggerated neuroinflammation (Fig. 6). These observations enhance our understanding about HMGB1-mediated immune response to CNS injury and infection, although the precise conditions for the induction of pro- versus anti- inflammation of HMGB1 remain unclear.

Regnase-1 locates at cytoplasm and distributes primarily in immune cells suggesting that this protein may be involved in immune regulation. Our data of microglia localization also supported this observation. As an mRNA endonuclease, Reganse-1 is critical for preventing severe autoimmune inflammatory diseases in mice by destabilizing cytokines mRNAs^24^. Regnase-1 also functions as a deubiquitinase to negatively regulate NF-κB and JNK inflammatory signaling pathways^48^. Moreover, it can protect mice from LPS-induced septic shock by inhibition of TLR4 signaling^49^ and reduce renal ischemia-reperfusion injury^50^. In our research, we demonstrated that Regnase-1 in microglia could be induced by HMGB1 treatment, while knockdown of Regnase-1 enhanced the production of proinflammatory cytokines and exacerbated neuronal injury, suggesting a novel physiological function of Regnase-1 in immune regulation. A negative regulatory feedback loop centered on Regnase-1 therefore is proposed to restrict the inflamed tissues in excessive inflammation state (Fig. 6). Further study with Regnase-1 gene knockout mice in our next work will strengthen the persuasion of this conclusion.

In our study, both mRNA and protein levels of Regnase-1 were very low in quiescent microglia. Stimulation by HMGB1 significantly promoted Regnase-1 expression both *in vitro* and *in vivo*. However, the underlying mechanism of HMGB1-induced Regnase-1 expression has not been elucidated. According to previous reports, MCP-1, LPS and IL-1β 25,26,27 induce the transcription of Regnase-1 through TLRs, NF-κB or MAPK pathways^21^,^28^. Whether the induction by HMGB1 shares the similar pathways is unclear and deserves further investigation.

HMGB1 preconditioning has been shown to be protective in several ischemia-reperfusion tissues, including kidney, heart and liver^51^,^52^,^53^,^54^. In parallel with current study, our ongoing study indicated that preconditioning with low dose recombinant HMGB1 induced ischemic tolerance in a rat model of focal cerebral ischemia-reperfusion (data not shown). As an HMGB1-inducible factor, Regnase-1 is likely to participate as a regulatory role of HMGB1-preconditioning. This interesting hypothesis is waiting for further verification.

In conclusion, this study suggests Regnase-1 serves as an HMGB1-inducible factor that limits HMGB1-mediated inflammation responses in microglia to avoid deleterious neuronal injury. Our discovery is helpful for better understanding of the immunopathology mechanism in inflammation-related CNS diseases.

## Methods

### Cell culture and HMGB1 treatment

BV2 cells and SH-SY5Y cells were maintained in Dulbecco’s Modified Eagle’s Medium (DMEM, Hyclone, Logan, Utah, USA) supplemented with 10% fetal bovine serum (FBS, BIOIND, Israel) and grown at 37 °C in a humidified environment (5% CO_2_, 95% air). Different time points (1–24 h) and doses (100–1,000 ng/ml) of pure recombinant HMGB1 (HMGBiotech, Italy) were added to medium for cell stimulus experiments. After treatment, RNA and protein from BV2 cells were obtained for further investigation. The conditioned media (CM) was collected for ELISA or neuron treatment.

### Regnase-1 gene knockdown in microglia

Regnase-1 knockdown siRNA (si-Zc3h12a, Invitrogen, Carlsbad, CA, USA) was transfected to BV2 cells by Lipofectamine to silence the gene expression. Briefly, BV2 cells were seeded on a 12-well plate until cell confluence reached about 80%. Lipofectamine 2000 (5 μl/ml, Invitrogen) and siRNA (2.5 μl/ml, Invitrogen) were mixed according to the manufacture’s instruction to form complex, added to serum and antibiotics-free DMEM medium and incubated with BV2 cells for 6 h. After replacement of fresh medium, the cells were continuously cultured for another 42 h. siRNA Negative Control (si-Control, Invitrogen) was employed. The sequence of si-Zc3h12a was shown in Table 1.

### Quantitative real-time PCR

Total cellular RNA was isolated from BV2 cells using TRIzol^®^ (Invitrogen) and reverse-transcribed to cDNA with the PrimeScript™ RT Reagent Kit (Takara, Japan) according to the datasheet from manufacturer. Gene products of IL-1β, IL-6, TNF-α, Regnase-1 and β-actin were then amplified by quantitative real-time PCR on ABI-Prism 7500 Real-Time PCR System (Applied Biosystems, Carlsbad, CA, USA) using SYBR^®^ Premix Ex TaqTM II (Takara, Japan). All gene-specific PCR products were normalized with the internal standard β-actin. The primer sequences are presented in Table 1.

### Western blot analysis

Total protein was extracted by Keygen Protein Extraction Kit (KEYGEN, China) according to the manufacture’s instruction. Denatured protein samples were resolved on SDS-PAGE and transferred to PVDF membranes (Millipore, Billerica, MA, USA). After blocking, the membranes were incubated at 4 °C overnight with primary antibodies including Regnase-1 (1:500, R&D, Minneapolis, MN, USA), IL-1β (1:500, Santa Cruz, Dallas, TX, USA), β-actin (1:1,000, ZSGB-Biology, China). Following washing, the membranes were then incubated with second antibodies including donkey anti-goat or goat anti-mouse HRP-conjugated secondary antibodies (1:5,000, Santa Cruz) for 2 h at room temperature (24 °C). Chemiluminescence detection performed with Kodak In-Vivo Imaging System F (Kodak, Rochester, NY, USA) was quantified and normalized to β-actin using ImageJ software (NIH, Bethesda, MD).

### ELISA

IL-1β level in BV2 CM was carried out using an IL-1β ELISA kit available from eBioscience (San Diego, CA, USA). Briefly, 96-well plate was coated with capture antibody overnight. After that, samples were added along with IL-1β antibody for 2 h with shaking. After rinsing, a secondary HRP-detection antibody was added for 45 min. Finally, wells were rinsed and substrate was added for 30 min incubation followed by the addition of stop solution. Plates were read by SpectraMax M5 (Molecular Devices, Sunnyvale, CA, USA).

### Cell viability assayed by CCK-8

Cells were seeded on 24-well plates at 2.5 × 10^5^ cells per well. After treatment, cells were incubated in 10% Cell Counting Kit-8 (CCK-8, Dojindo; Kumamoto, Japan) at 37 °C for 1 h. Cell viability was determined by SpectraMax M5 (Molecular Devices) with the absorbance wavelength of 450 nm.

### Annexin V-FITC and PI staining

The percentage of apoptotic cells in supernatants collected from SH-SY5Y was measured using Annexin V-FITC Apoptosis Detection Kit I (BD Biosciences, San Diego, CA, USA) as per the manufacturer’s instructions. Briefly, cells were collected (detached by 0.25% trypsinization without EDTA) and resuspended in 100 μl of 1 × Binding Buffer. Then, 5 μl Annexin V-FITC and 10 μl propidium iodide (PI) were added to the cell suspension in dark for 15 min. After that, 400 μl of 1 × Binding Buffer was added to each tube. BD FACSAria^TM^ IIu (BD Biosciences) was used for fluorescence acquisition and data were analyzed with FACSDiva software (BD Biosciences).

### Animals, treatment and tissue preparation

Wistar male rats (180~200 g) were purchased from the Animal Center of Southern Medical University. All animal experiments were carried out in accordance with the Guidelines for Animal Care and Use of the Southern Medical University. All experimental protocols were approved by the animal care committee of the Nanfang Hospital which is affiliated with Southern Medical University. All efforts were made to minimize animal suffering and to reduce the number of animals used. There were no ethic issues during our experiments.

The recombinant HMGB1 (8 μg/kg) was injected into the right lateral ventricle of rats with microinjection syringe using the following coordinates: 0.8 mm posterior to the bregma, 2.0 mm lateral to the midline, and 3.5 mm under the dura mater. Rats in control group were received same volume of vehicle saline. After 24 h, animals were anesthetized and transcardially perfused with phosphate buffer solution (PBS). The brains were then isolated and fixed with cold 4% paraformaldehyde. Serial brain sections (6 μm thick) were collected for further analysis.

### Immunohistochemistry

Brain sections were sliced, fixed with 4% formaldehyde solution, washed and permeabilized with 0.5% Triton X-100 (MP Biomedicals, CA, USA) in PBS followed by incubation with H_2_O_2_ for 10 min. A Biotin-Streptavidin HRP Detection System (ZSGB-Biology, China) was used to detect Regnase-1 expression. Sections were incubated with blocking buffer containing goat serum for 1 h at room temperature followed by addition of antibody to Regnase-1 (1:200; R&D) and incubated overnight at 4 °C. After washing, sections were incubated with biotinylated goat anti-mouse IgG (1:200) for 1 h and incubated with horseradish peroxidase (HRP) conjugated streptavidin for 15 min. HRP reaction product was incubated with 3, 3′-diaminobenzidine (ZSGB-Biology) for 1 min. Haematoxylin staining (ZSGB-Biology) for 5 min revealed cell nucleus.

### Immunofluorescence

Brain sections were fixed with 4% formaldehyde solution, incubated with 0.1% Triton X-100 for 30 min, then blocked with 5% bovine serum albumin (MP Biomedicals, Santa Ana, CA, USA) in PBS for 30 min. Samples were incubated at 4 °C overnight with anti-Iba1 antibody (1:200; Abcam, Cambridge, UK) combined with anti-Regnase-1 antibody (1:200; R&D). Then they were washed three times with PBS and incubated with secondary antibodies conjugated with Alexa^®^ Fluor 488 or 647 (1:500, Abcam) at room temperature for 2 h. The slides were mounted in prolong gold anti-fade reagent with DAPI (Invitrogen) for 2 h and observed with a confocal laser scanning microscope (OLYMPUS FV10C-W3, Tokyo, Japan).

### Statistical analysis

The values were presented as mean ± standard deviation (SD). Multiple comparisons were analyzed by either one-way ANOVA or two-way ANOVA as appropriate. For two groups designed experiments, comparisons were determined by unpaired Student’s t-test. Statistical analysis was performed in the SPSS 20.0 statistical program (SPSS, Chicago, IL, USA). *P* < 0.05 was considered to be statistically significant in the compared group.

## Additional Information

**How to cite this article**: Liu, X.-X. *et al*. Regnase-1 in microglia negatively regulates high mobility group box 1-mediated inflammation and neuronal injury. *Sci. Rep.* **6**, 24073; doi: 10.1038/srep24073 (2016).

## Supplementary Material

Supplementary Information

## Figures and Tables

**Table 1:** The sequences of siRNA and primers.

Primers and siRNA	Sequences
si-Zc3h12a	5′-CCUGGACAACUUCCUUCGUAAGAAA-3′
β-actin	Forward: 5′-CCAAAAGATGAAGGGCTGCTT-3′ Reverse: 5′-GAAAAGAAGGTGCTCATGTCCTC-3′
Regnase-1	Forward: 5′-CAATGTGGCCATGAGCCAT-3′ Reverse: 5′-AGTTCCCGAAGGATGTGCTG-3′
IL-1β	Forward: 5′-CCAAAAGATGAAGGGCTGCTT-3′ Reverse: 5′-GAAAAGAAGGTGCTCATGTCCTC-3′
IL-6	Forward: 5′-TCTTGGGACTGATGCTGGTG-3′ Reverse: 5′-TGCCATTGCACAACTCTTTTCT-3′
TNF-α	Forward: 5′- TCTCATTCCTGCTTGTGGCA-3′Reverse: 5′- GGTGGTTTGCTACGACGTGG-3′
