# Supplementary material for: Regnase-1 in microglia negatively regulates high mobility group box 1-mediated inflammation and neuronal injury
Source: Sci Rep. 2016 Apr 5;6:24073. doi: 10.1038/srep24073 (PMC4820713; doi:10.1038/srep24073)
Supplement: Supplementary Information [file srep24073-s1.pdf]

Supplementary information for

# Regnase-1 in microglia negatively regulates high mobility group box 1-mediated inflammation and neuronal injury

Xiao-Xi Liu [1], Chen Wang [1,2], Shao-Fei Huang [1], Qiong Chen [1], Ya-Fang Hu [1], Liang Zhou [1,*], Yong Gu [1,*]

[1] Department of Neurology, Nanfang Hospital, Southern Medical University. Guangzhou, Guangdong 510515, P. R. China.

[2] Department of Neurology, the Second Affiliated Hospital & Yuying Children's Hospital, Wenzhou Medical University. Wenzhou, Zhejiang 325000, P. R. China.

* **Corresponding author:** Yong Gu, Department of Neurology, Nanfang Hospital, Southern Medical University. 1838# Guangzhou Avenue North, Guangzhou, Guangdong 510515, P. R. China. Tel: +86-20-61641964. E-mail: yonggu@smu.edu.cn.

* **Corresponding author:** Liang Zhou, Department of Neurology, Nanfang Hospital, Southern Medical University. 1838# Guangzhou Avenue North, Guangzhou, Guangdong 510515, P. R. China. Tel: +86-20-61641964. E-mail: zhouliang_1963@126.com.

Supplementary Figure S1 is related to Figure 2a.
Supplementary Figure S2 is related to Figure 3a
Supplementary Figure S3 is related to Figure 4e.

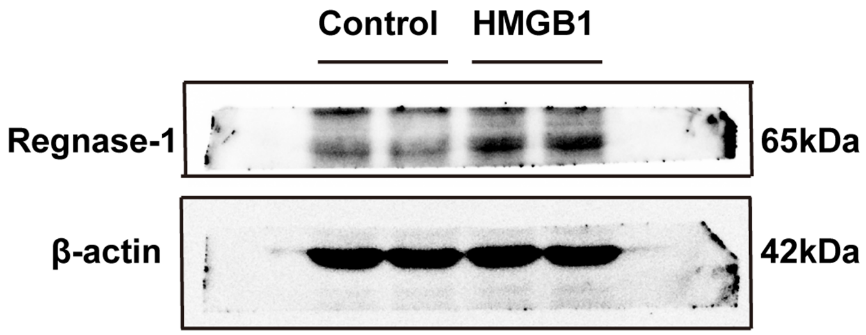


**Supplementary Figure S1** Full-length image of Western blot in Figure 2a. Regnase-1 protein expression in BV2 cells with or without HMGB1 (1,000g/ml) treatment was measured by Western blot. β-actin was used as internal loading control.

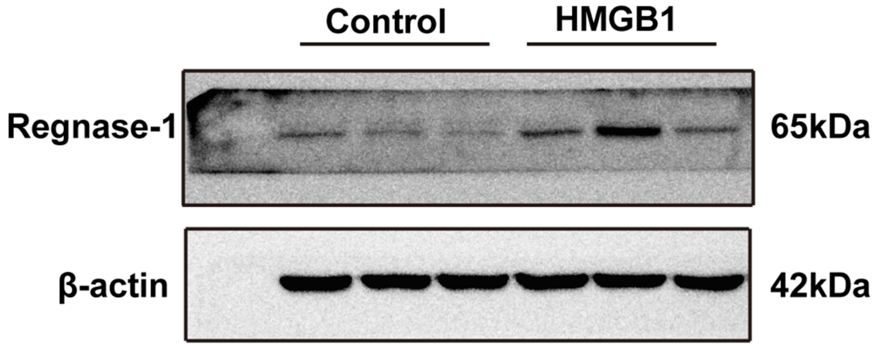


**Supplementary Figure S2** Full-length image of Western blot in Figure 3a. Rats were randomly divided into two groups and treated with either saline (Control) or HMGB1 (8 μg/kg) for 24 h. Regnase-1 protein levels in the brain of rats were determined by Western blot. β-actin was used as internal loading control.

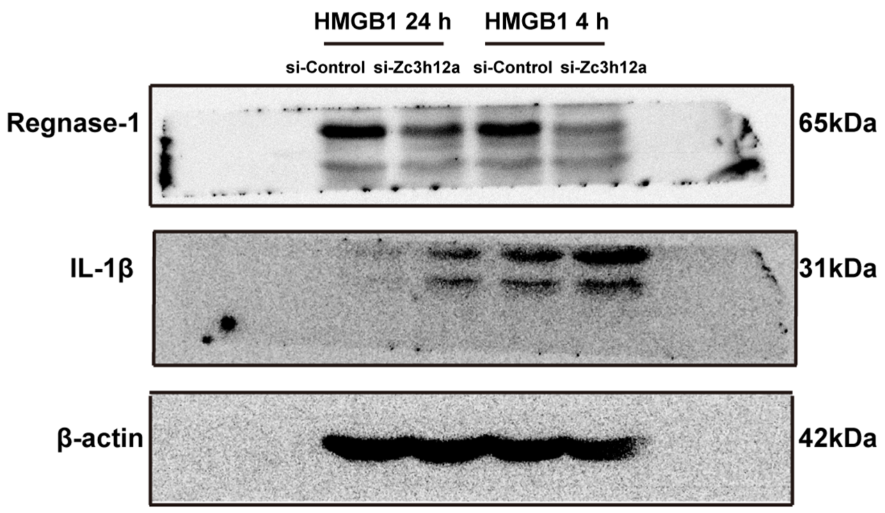


**Supplementary Figure S3** Full-length image of Western blot in Figure 4e. BV2 cells transfected with si-Control or si-Zc3h12a were treated with HMGB1 (1,000 ng/ml) for 4 h or 24 h. The protein levels of Regnase-1 and IL-1β were evaluated by Western blot. β-actin was used as internal loading control.
